# Assessment of Screening for Adverse Childhood Experiences and Receipt of Behavioral Health Services Among Children and Adolescents

**DOI:** 10.1001/jamanetworkopen.2022.47421

**Published:** 2022-12-19

**Authors:** Sonya Negriff, Mercie J. DiGangi, Margo Sidell, Jialuo Liu, Karen J. Coleman

**Affiliations:** 1 Department of Research & Evaluation, Kaiser Permanente Southern California, Pasadena; 2 Kaiser Permanente Bernard J. Tyson School of Medicine, Pasadena, California; 3 Department of Pediatrics, Kaiser Permanente Southern California, Bellflower

## Abstract

**Importance:**

Screening for adverse childhood experiences (ACEs) in primary care settings has been increasing as a response to the overwhelming and consistent evidence of the deleterious associations between ACEs and later physical and mental health. However, there is little empirical guidance on the appropriate implementation of ACEs screening in pediatric primary care.

**Objective:**

To test the use of a pilot intervention for ACEs screening and referral on the receipt of behavioral health care for children and adolescents within a large integrated health care delivery system.

**Design, Setting, and Participants:**

A retrospective cohort study was conducted in a large integrated health care system serving Southern California. Child and adolescent members of the target health care system younger than 18 years between July 1, 2018, and November 30, 2021, who received a positive screening for ACEs at the pilot clinic were included. This pilot clinic implemented an intervention that included additional screening questions and incorporated social workers into the process of evaluation and referral for behavioral health needs following ACEs screening.

**Exposures:**

ACEs screening.

**Main Outcomes and Measures:**

Visit to a behavioral health care service within 90 days of a positive ACEs screen determined as a score of 1 or higher and behavioral symptoms.

**Results:**

The cohort consisted of 4030 children (mean [SD] age, 9.94 [4.55] years) with positive ACEs screening, 48% adolescents (11-17.99 years), approximately equal gender (51% females), 73% Hispanic, and 33% with Medicaid insurance. After the intervention, children were more likely to have a behavioral health services visit within 90 days of the screening than before the intervention (from 4.33% to 32.48%; incidence rate ratio, 7.50; 95% CI, 1.55-36.2).

**Conclusions and Relevance:**

In this cohort study, the implementation of a new ACEs screening and referral process was associated with increased receipt of behavioral health services among children with a positive ACEs screening. This could be useful strategy for other health care systems responding to state and local mandates to screen and provide care for children with ACEs.

## Key Points

**Question:**

How do large health care systems address the behavioral health needs of children with positive adverse childhood experiences (ACEs) screening?

**Findings:**

In this cohort study including 4030 children and adolescents, a pilot individualized ACEs screening and referral process was conducted. Findings showed an association with an increase in the rate of visits to behavioral health care services compared with usual care.

**Meaning:**

As ACEs screening use becomes more common, more studies are warranted across different types and sizes of health care delivery systems to give clinicians evidence-based guidance on the screening and referral process that may function best for their circumstances.

## Teaser

This cohort study assesses the association of a pilot intervention for screening children and adolescents for adverse childhood experiences with receipt of behavioral health care in a large integrated health system.

## Introduction

There is overwhelming and consistent evidence of the deleterious associations between adverse childhood experiences (ACEs) and later physical and mental health^1^; however, there are a number of challenges with wide-scale screening and care provision based on the results in health care settings.^2^ These challenges are due, in part, to the slow uptake of ACE screening in pediatric primary care^3^ and few established guidelines for referral to services and follow-up based on the results of the screening. In addition, there is some suggestion that until there are evidence-based guidelines for referral and care, screening for ACEs may do more harm than good.^4^ This is balanced with the opportunity for educating parents about how life experiences may impact physical and mental health, as well as providing more individualized treatment for any child with a positive ACEs screening.^5,6^

Despite the challenges identified for ACEs screening and provision of care, at least 37 states have enacted some form of legislation to prevent or reduce ACEs.^7^ Most state mandates are focused on the establishment of workgroups/taskforces or trauma-informed education programs without a consideration of how health care systems would identify children needing services.^7^ In 2020, California became the first state to mandate statewide ACEs screening for patients with Medi-Cal/Medicaid (hereafter Medicaid), and the state provided funding to reimburse clinicians for ACEs screening.^8^ In addition, a set of guidelines for screening cutoffs and treatment was published with recommendations for referrals to behavioral health services as the primary treatment for ACEs.^8^

To our knowledge, only a few studies have reported the implementation of ACEs screening and treatment response among pediatric primary care patients.^9^ While these studies report high feasibility and acceptability of ACEs screening from both patients and clinicians, to date, none have provided data on the results of the screening as recommended by the published guidelines (ie, referral to behavioral health services) nor have they provided details about the workflows necessary to implement the guidelines in large health care settings serving millions of families.

The present study was designed to address these limitations by evaluating whether a pilot intervention implemented in pediatric primary care of a large integrated health care system was associated with an increase in the rate of completed visits to behavioral health services within 90 days after a positive ACEs screening.

## Methods

### Setting and Study Population

The study was conducted in an integrated health care system serving more than 4.7 million members, including approximately 1.5 million children. The evaluation of the ACEs clinical initiative was determined as exempt by the institutional review board of Kaiser Permanente Southern California with a waiver of informed consent due to the impossibility of obtaining consent from thousands of patients. The study was reported in accordance with the Strengthening the Reporting of Observational Studies in Epidemiology (STROBE) reporting guideline.^10^

The data for the present study were obtained from the electronic health records of children and adolescents younger than 18 years who were members of the health care system between July 1, 2018, and November 30, 2021, and completed ACEs screening at the pilot clinic. The pilot clinic located in Los Angeles County is staffed by 35 pediatricians, and had between 1500 and 2000 monthly pediatric primary care visits eligible for ACEs screening during the study period.

### Design and Intervention

The study design was a single group interrupted time series—a strong evaluation design when randomization is not possible.^11^ This design allows for the evaluation of an intervention with clearly defined preintervention and postintervention periods that is expected to change a given outcome, assuming that without the intervention, the preintervention trend would continue unchanged through the postintervention period.^11^

**Figure 1.**
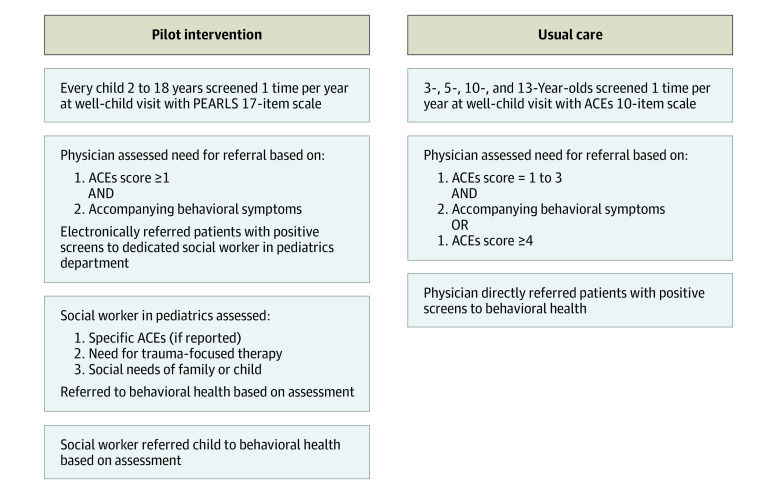
Usual Care vs Pilot Intervention Screening and Referral Process ACEs indicates adverse childhood experiences; PEARLS, Pediatric ACEs and Related Life-Events Screener.

Before the intervention, the usual practice of the pilot clinic (baseline period) used the original Kaiser Permanente–Centers for Disease Control and Prevention ACEs questionnaire with wording adapted from the Center for Youth Wellness ACE-Q questionnaire.^12^ Parents of children aged 2 to 12 years completed the questionnaire for them, and adolescents aged 13 years and older completed it for themselves. Score cutoffs for referral aligned with the guidelines of ACEs Aware^8^ including direct referral from the pediatrician to behavioral health services for an ACEs score of 4 or higher or ACEs score of 1 to 3 with accompanying behavioral or mental health symptoms. The workflow (described previously^13^) is shown in Figure 1.

The intervention was implemented in February 2021 at the pilot clinic (intervention workflow shown in Figure 1). There were several notable changes from usual care: (1) change in screener (from ACEs to Pediatric ACEs and Related Life-Events Screener [PEARLS]),^14^ (2) referral from the pediatrician to a medical social worker for positive screens, (3) and warm handoff (ie, direct connection) from the social worker to behavioral health services after assessment. These changes were made based on lessons learned from the usual care screening process, feedback from pediatricians and behavioral health professionals, and policy changes in California necessitating the use of PEARLS screener for Medicaid reimbursement. PEARLS included the 10 usual care screening questions plus 7 questions assessing neighborhood/community violence exposure, discrimination, housing instability, food insecurity, separation from parent due to foster care or immigration, parent/guardian death, and parental serious physical illness or disability. While the score on part 1 was used for Medicaid reimbursement, the score on both parts was used to determine a positive screen. The pediatrician referred any child with a positive screen (score ≥1 and behavioral and/or mental health symptoms) to a medical social worker and the social worker completed a psychosocial assessment to identify service needs including behavioral health, parenting classes, food insecurity, and housing insecurity. If a referral to behavioral health services was needed, the social worker provided a warm handoff by directly connecting the family to behavioral health services via telephone. Pediatricians and the pediatric social worker assigned to this clinic were trained on the new process.

### Outcome

The main outcome of the evaluation was the rate of completed visits to behavioral health services within 90 days after a positive ACEs screening. All screening and visit data were obtained from the electronic health records.

### Covariates

Several covariates from the electronic health records were included in the analyses based on the literature in this area^15,16^ and interest in the findings from a health care system implementation standpoint. These included age at screening (2-5 years, 6-10 years, and 11-17.99 years), gender (male, female), race and ethnicity (Asian/Pacific Islander, Black, Hispanic, White, and other/unknown), and Medicaid status (yes/no). Data on race and ethnicity were obtained from membership enrollment data and included due to differences in ACEs prevalence.

### Statistical Analysis

Poisson distribution models using the single group interrupted time series design were applied to assess whether the intervention increased the rate of completed visits to behavioral health services among those with positive ACEs screening (score ≥1). The model included month as the time variable, a dummy variable for the intervention indicating whether a specific time point was before (preintervention) or after (postintervention) the February 2021 implementation, and the interaction of month by intervention. Fully adjusted models further included age group, race and ethnicity, gender, and Medicaid status. A random effect of child was included in all models to account for children who had multiple ACEs screenings. In addition to the main analyses, we conducted the same models described above but with the ages of the intervention site restricted to 3, 5, 10, and 13 years to match the usual care procedures. These models did not differ meaningfully, so we only report the results for all ages. All statistical tests were 2-sided, and *P* < .05 was the significance threshold. Data analysis was conducted with R, version 4.0.4 (R Foundation for Statistical Computing).

## Results

**Figure 2.**
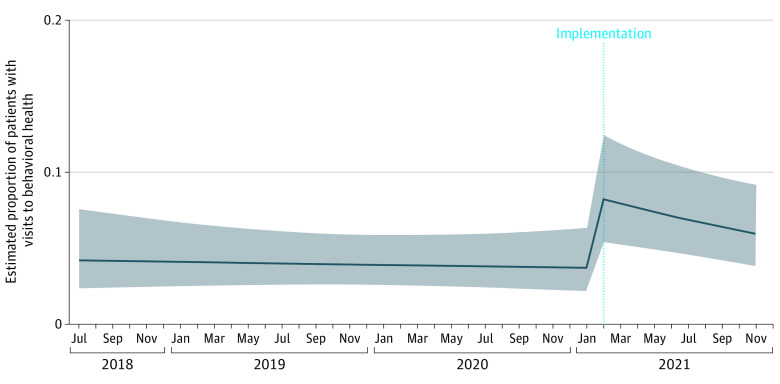
Interrupted Time Series Showing the Rate of Visits to Behavioral Health Services Preintervention and Postintervention Solid line indicates estimated proportion of patients with visits to behavioral health services; shaded area, 95% CI.

The cohort consisted of 4030 children with positive ACEs screening from 4332 encounters (some children had multiple records). As shown in Table 1, the mean (SD) age of the study population was 9.94 (4.55) years, 48% were adolescents (11-17.99 years), gender was approximately equal (51% girls, 49% boys), 73% were Hispanic, and 33% had Medicaid insurance. Of all positive screens in the analysis cohort (n = 4332), 1383 occurred during the preintervention period and 2949 in the postintervention period (Table 2). As shown in Figure 2 and Table 2, there was a significant increase in the rate of completed visits to behavioral health services after the implementation of the intervention. Postintervention, children whose screening was positive were 7.5 (95% CI, 1.55-36.2) times more likely to have a behavioral health visit than before (during usual care), which was maintained over time. This was reflected in adjusted rates of 4.33% preintervention to 32.48% postintervention. This differs from the rates shown in Figure 2 due to all covariates set to the reference category in the figure. For the covariates, older children (ages 6-10 and 11-17 years compared with 0-5 years) and girls were more likely to have a behavioral health visit across the entire study period (Table 2).

**Table 1.** Characteristics of Study Sample

Characteristic	Pilot clinic, No. (%)
No.	4030
Age, mean (SD), y	9.94 (4.55)
Age group, y	
2-5	1116 (28)
6-10	985 (24)
11-17.99	1929 (48)
Gender	
Male	1962 (49)
Female	2068 (51)
Race and ethnicity	
Asian/Pacific Islander	146 (4)
Black	545 (14)
Hispanic	2941 (73)
White	254 (6)
Other/unknown^a^	144 (4)
Medicaid status, yes	1338 (33)

^a^ Other race and ethnicity includes Native American, Native Alaskan, multiple races and ethnicities, and other.

**Table 2.** IRRs and 95% CIs From Fully Adjusted Interrupted Time Series Analyses

Characteristic	Analysis cohort, No. (%)^a^		Intervention clinic	
	Pre	Post	IRR (95% CI)	*P* value
Month	NA	NA	1.00 (0.97-1.02)	.80
Intervention				
Pre	1383 (100)	0	1 [Reference]	
Post	0	2949 (100)	7.50 (1.55-36.2)	.01
Age group, y				
0-5	738 (53.36)	478 (16.21)	1 [Reference]	
6-10	326 (23.57)	773 (26.21)	2.04 (1.51-2.77)	<.001
11-17.99	319 (23.07)	1698 (57.58)	2.90 (2.19-3.83)	<.001
Gender				
Male	688 (49.75)	1420 (48.15)	1 [Reference]	
Female	695 (50.25)	1529 (51.85)	1.22 (1.04-1.43)	.02
Race and ethnicity				
Asian/Pacific Islander	70 (5.06)	206 (6.99)	0.43 (0.23-0.82)	.01
Black	52 (3.76)	130 (4.41)	0.70 (0.47-1.04)	.08
Hispanic	185 (13.38)	402 (13.63)	0.98 (0.71-1.35)	.90
White	31 (2.24)	92 (3.12)	1 [Reference]	
Other/unknown^b^	1045 (75.56)	2119 (71.85)	1.13 (0.67-1.91)	.60
Medicaid	499 (36.08)	951 (32.25)	1.02 (0.86-1.21)	.80
Month × postintervention	NA	NA	0.97 (0.93-1.01)	.14

Abbreviations: ACEs, adverse childhood experiences; IRR, incidence rate ratio; NA, not applicable.

^a^ Analysis cohort: 4332 records (pre, 31.93%; post, 68.07%) from 4030 children (some children have multiple records) with ACES identified in primary care.

^b^ Other race and ethnicity includes Native American, Native Alaskan, multiple races and ethnicities, and other.

## Discussion

The present study evaluated whether an intervention was associated with improvement in the receipt of appropriate services for children and adolescents with positive ACEs screening. Overall, the results showed that the pilot intervention, which included additional screening questions and social work assessment, was associated with an increase in the rate of completed behavioral health visits, from 4.33% to 32.48% based on the adjusted model (7.5 times increase; 95% CI, 1.55-36.2) and did not significantly decline postintervention. Cross-sectional data from 3 US national surveys indicate that 41% of children aged 2 to 9 years and 62% of adolescents aged 10 to 17 years with high ACEs and high psychological distress symptoms did not have any clinical contact with behavioral health services.^17^ Changes to the ACEs screening and referral processes that were used in our pilot intervention could substantially increase the rates of service provision for these at-risk youth.

Although it is difficult to determine exactly what part of the pilot intervention was responsible for the increase—the additional screening questions or the subsequent assessment by the social worker—it is likely due to the work of the social worker who completed a psychosocial assessment and a warm handoff to behavioral health services if a referral was indicated. This is supported by evidence that care management by social workers can improve the use of pediatric mental health services.^18^ However, very few studies have reported on the full process of assessment, referral, and treatment associated with positive ACEs screening.^9^ Of those that have reported on the continuum of care, one study^19^ used a similar approach to the current study and used wellness navigators to address food insecurity and enroll families in parenting classes in a community clinic setting.^19,20^ They found that these wellness navigators initiated services for 95% of the referrals made and that medical professionals and caregivers deemed the navigators as crucial for improving the quality of medical care. In our health care system, pediatric social workers often function as care navigators, giving referrals to a variety of internal and community resources. They may also function as gatekeepers to behavioral health, ensuring that only the appropriate referrals are made.

Of the covariates, we found that children aged 11 to 17 years had the highest rate of visits to behavioral health services across the study period. This is not unexpected as national data show that 1 in 4 adolescents aged 12 to 17 years reported receipt of mental health services in the past year.^21,22^ Girls were at a slightly higher likelihood of behavioral health visits, which also aligns with higher rates of mental health services in females than males in the US.^23^ Similarly, ACE-exposed female college students were found to report more mental health symptoms than males.^24^ Taken together, this suggests that females may be at higher risk for developing symptoms as a result of ACEs and may have higher need for behavioral health treatment. Our results did not indicate any racial or ethnic disparities, although others have found that non-Hispanic Black children with high ACEs and/or psychological distress had less contact with behavioral health services than non-Hispanic White children,^17^ and higher ACE scores have been found to be associated with more harmful mental health for racial and ethnic minority individuals.^25^ Conversely, a previous longitudinal study found that the impact of ACEs on mental health was greater for non-Hispanic White than Black or Hispanic adults.^26^ This may be related to lower rates of health care access for non-Hispanic Black children with ACEs.^27^ It is not clear from this study whether our lack of disparities is accurate or due to less access or lower acceptance of referrals for Black or Hispanic families. Our health care system actively works to reduce disparities,^28,29^ and that may be reflected in our data as well.

### Limitations

There are a number of limitations to be considered when interpreting these findings. First, the data came from one region (Southern California) of a large integrated health care system. Rates of visits to behavioral health services as a result of ACEs screening may differ based on geographic differences and population characteristics that may alter ACEs prevalence. Additionally, in populations that have higher positive screening rates, there will be greater demand for services, and the rates of visits with behavioral health services will be contingent on the availability of clinicians.^30^ We only captured visits that occurred within our health care system. Any services provided by external behavioral health professionals are not reflected in the data. Lastly, these data cannot be used to determine whether the referral to behavioral health services was appropriate and whether receipt of services actually reduced mental health symptoms in those referred children. These critical issues could be investigated in future studies as more data accumulate on longer-term outcomes.

## Conclusions

The findings of this study suggest that an intervention that included the use of medical social workers in the ACEs screening and referral process was associated with an increase in the receipt of behavioral health services among children with positive ACEs screening. We cannot infer from these findings that the child received better or more relevant care. The implementation of this updated workflow occurred during the COVID-19 pandemic, essentially requiring the social work visit to be a virtual visit. This provides evidence that this type of assessment and referral process can be handled remotely and does not necessitate an on-site social worker. This is an important point for feasibility for health care professionals who seek to implement ACEs screening. Additional components of feasibility of ACEs screening include health care professionals’ receipt of adequate training, streamlined workflows, and availability of behavioral health resources.^31^ As ACEs screening continues to gain traction, more studies are needed across different types and sizes of health care delivery systems to give clinicians evidence-based guidance on the screening and referral process that may function best for their circumstances.

## Notes

Supplement.
Data Sharing Statement
