# Supplementary material for: Assessment of Screening for Adverse Childhood Experiences and Receipt of Behavioral Health Services Among Children and Adolescents
Source: JAMA Netw Open. 2022 Dec 19;5(12):e2247421. doi: 10.1001/jamanetworkopen.2022.47421 (PMC9857176; doi:10.1001/jamanetworkopen.2022.47421)
Supplement: Supplement. — Data Sharing Statement [file jamanetwopen-e2247421-s001.pdf]

Data Sharing Statement

Negriff. Assessment of Screening for Adverse Childhood Experiences and Receipt of Behavioral Health Services Among Children and Adolescents. *JAMA Netw Open*. Published December 19, 2022. doi:10.1001/jamanetworkopen.2022.47421

Data
Data available: No

Additional Information
Explanation for why data not available: Data sharing is not possible because of the sensitive nature of this data.
